# A case of secondary syphilis presenting like pemphigus with positive direct immunofluorescence

**DOI:** 10.1016/j.jdcr.2023.09.023

**Published:** 2023-10-04

**Authors:** Caroline J. Stone, Lowell Nicholson, Scott R. Florell, Mazdak A. Khalighi, Bethany K.H. Lewis

**Affiliations:** a University of Utah School of Medicine, Salt Lake City, Utah; b Department of Dermatology, University of Utah, Salt Lake City, Utah; c Department of Dermatopathology, University of Utah, Salt Lake City, Utah

**Keywords:** direct immunofluorescence, lues maligna-type secondary syphilis, pemphigus-like secondary syphilis, pemphigus vulgaris, syphilitic pemphigus

## Introduction

Syphilis is commonly mistaken for other disease processes and diagnosis is often delayed. Cutaneous manifestations are highly variable and include maculopapular eruptions, palmoplantar lesions, mucoid plaques (“condyloma lata”), and alopecia.^1^ Here, we report a singular case of an adult male with both secondary syphilis and neurosyphilis presenting with unique pemphigus-like lesions, mucosal involvement, and, remarkably, positive staining on direct immunofluorescence (DIF).

## Case report

A male in his 60s presented to dermatology clinic with a diffuse rash of 2 months’ duration. He reported that his rash initially started on the forehead but subsequently spread to involve the torso and extremities, sparing the palms and soles. He additionally reported fatigue, arthralgias, and blurry vision. He denied any fever, chills, night sweats, weight loss, difficulty eating or drinking, or urinary symptoms. Social history was notable for multiple male sexual partners over the last several years but none within the prior 12 months. He denied any history of HIV or other sexually transmitted infections.

**Fig 1:**
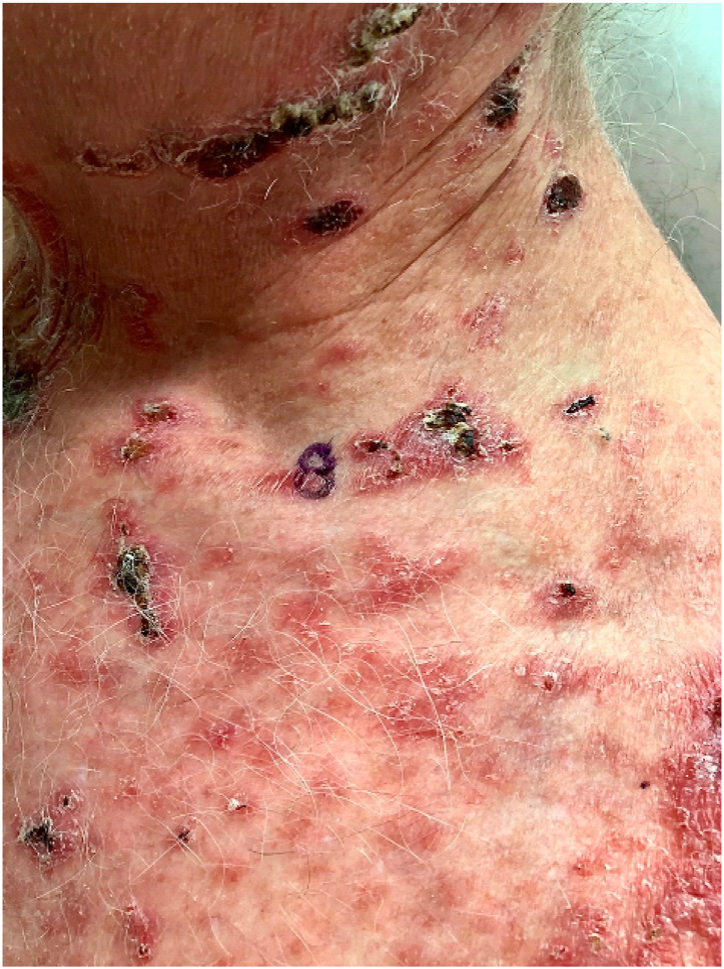
Erosion of the lip and erythematous hemorrhagically crusted papules coalescing into plaques.

**Fig 2:**
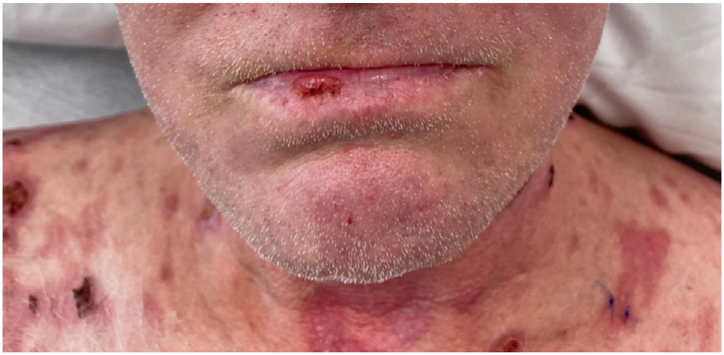
Erosion of the lip and erythematous hemorrhagically crusted papules coalescing into plaques.

**Fig 3:**
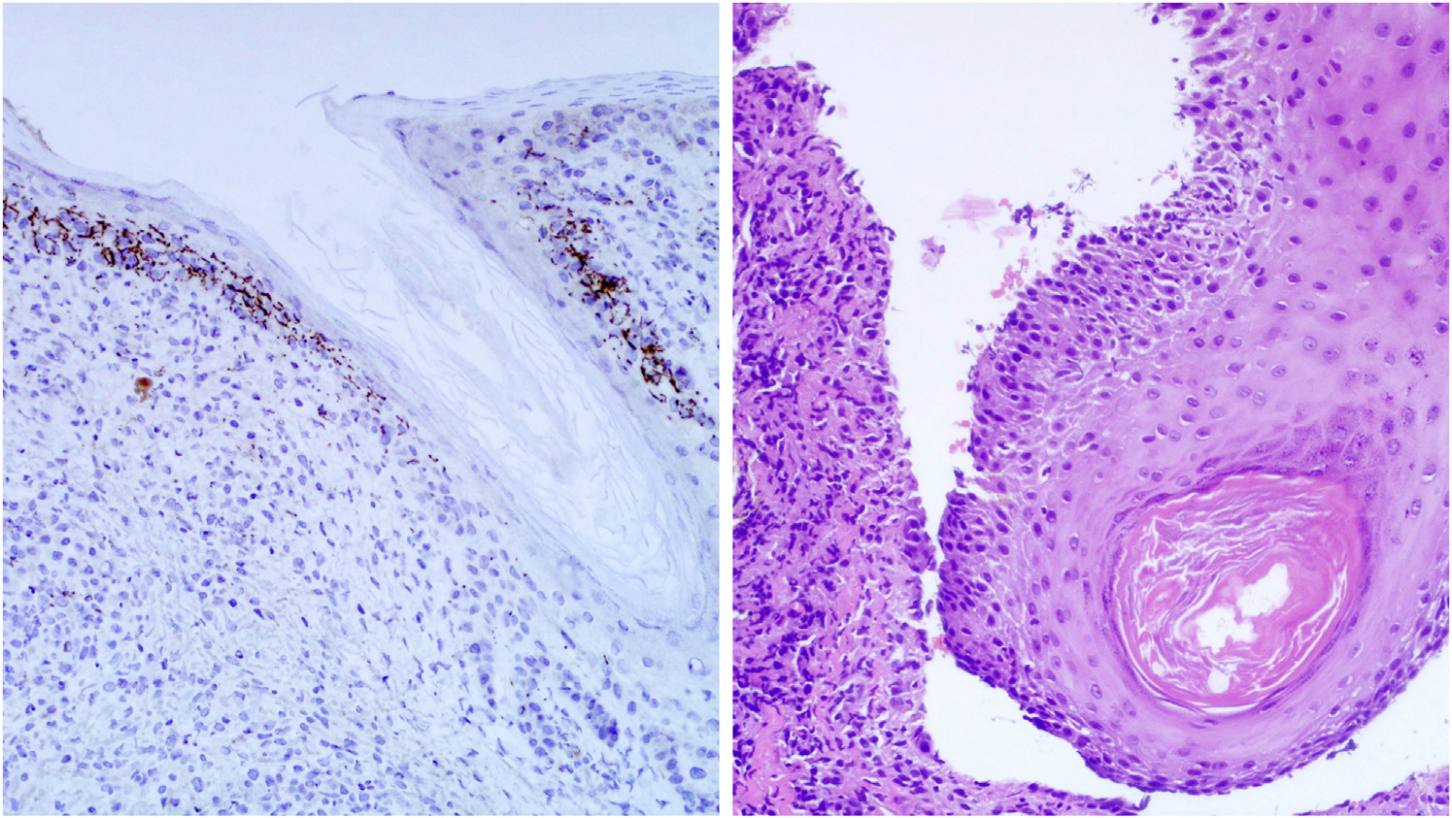
On the *left*, a *Treponema pallidum* immunostaining demonstrates numerous spirochetes along the dermal-epidermal junction, around blood vessels, and within the dermal inflammatory milieu. Approximately 60% of the lymphocytes are CD3+ T-cells and 40% are CD20+ B-cells. Kappa and lambda by in situ hybridization show a polytypic plasma cell infiltrate. On the *right*, leveled sections of a bisected punch demonstrate parakeratosis with inflammatory cell debris overlying an epidermis manifesting focal intraepithelial acantholysis that also involves the follicle. There is a superficial and deep perivascular and interstitial, somewhat nodular infiltrate composed of lymphocytes with large numbers of plasma cells and a few scattered eosinophils. There are also histiocytes and neutrophils.

**Fig 4:**
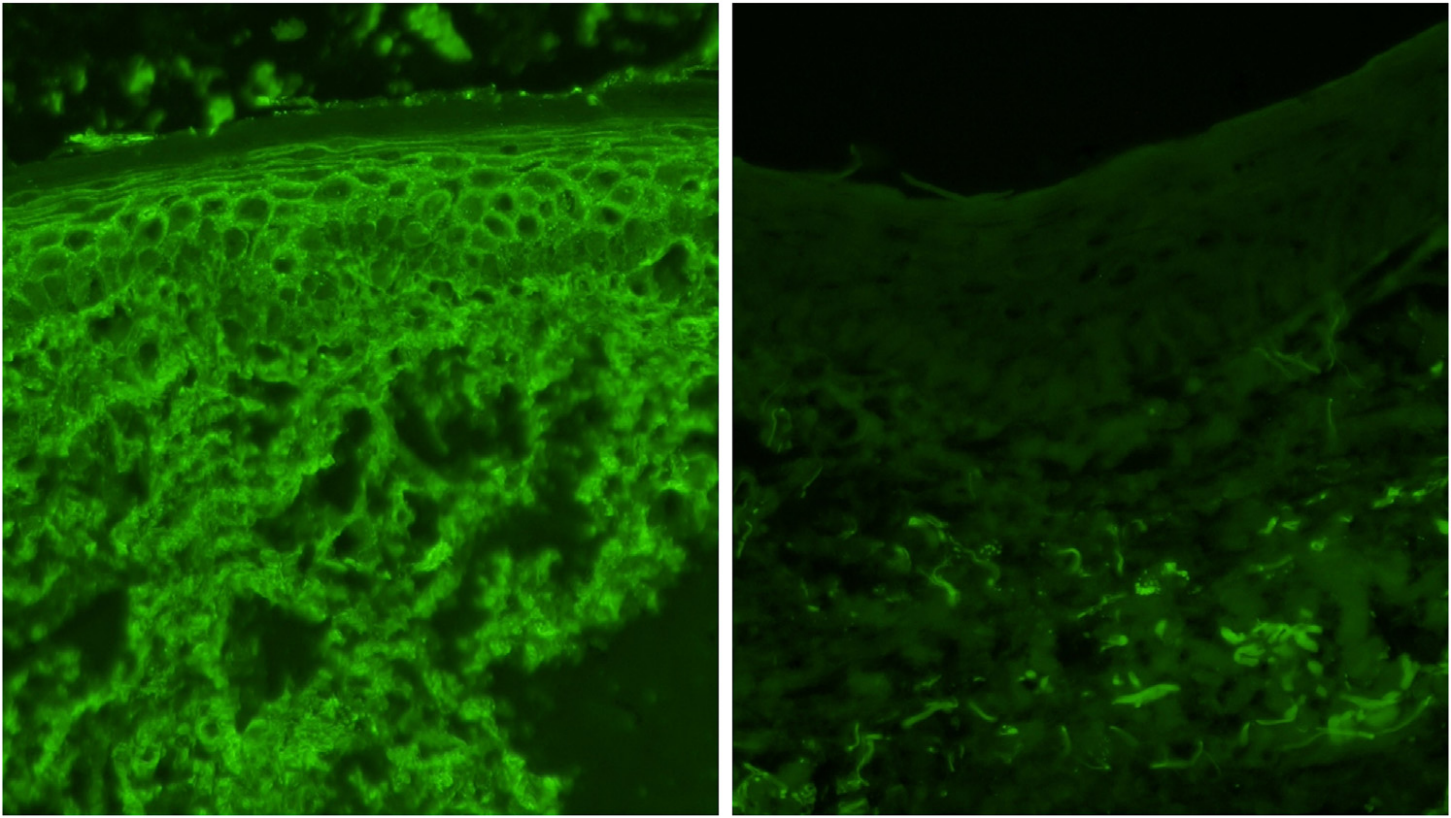
IgG staining 400× on the *left* and negative control on the *right*.

On physical examination, there were multiple eroded, crusted erythematous papules and larger plaques scattered on the scalp, face, neck, torso, and upper extremities (Fig 1). Examination of the mucocutaneous surfaces revealed a superficial erosion on the right lower mucosal lip (Fig 2). Patient was noted to have injected sclera. Initial diagnostic work up was notable for hyponatremia (serum sodium 125 mmol/L) with normal kidney function, mildly elevated aspartate aminotransferase (47 U/L), markedly elevated inflammatory markers (erythrocyte sedimentation rate 93, C-reactive protein 6.5), and a positive rapid plasma reagin with a titer of 1:1024. A punch biopsy was performed and showed acantholysis with subsequent positive immunostaining for *Treponema pallidum* (Fig 3). DIF was notable for granular IgG on the surface of epithelial cells that was continuous in multiple areas but seldom covered the entire epithelial cell surface (Fig 4). Indirect immunofluorescence/enzyme-linked immunoassay testing was not pursued once serologic confirmation of syphilis was obtained.

Taken together, these findings confirmed a diagnosis of lues maligna-type secondary syphilis. Out of concern for neurosyphilis with possible ocular involvement, a lumbar puncture was performed, which showed an elevated white blood cell of 9 × 10^9^/L in his cerebrospinal fluid. The patient was treated with penicillin G 24 million units per day administered for 14 days, resulting in complete resolution of his rash and visual symptoms.

## Discussion

Due to the heterogeneity of its clinical presentation, diagnosing syphilis can be challenging. Our patient presented with widely distributed eroded and crusted erythematous papules and plaques with mucosal involvement strikingly similar to pemphigus variants. Although rarely reported, a vesiculopustular form of syphilis termed “syphilitic pemphigus” has been described. In the literature, this form of syphilis typically presents with bullae and pustules and involves the palms and soles; DIF has not been noted to be positive in these cases. In contrast, our patient had no palm or sole involvement but did have a positive DIF.

The autoimmune blistering disease pemphigus is diagnosed by DIF of a perilesional biopsy. It detects the deposition of IgG and/or complement component 3 at the keratinocyte cell membrane with serum antidesmoglein 1 and/or antidesmoglein 3 antibodies by enzyme-linked immunoassay.^2^ In cases of pemphigus vulgaris, DIF is positive in 90% to 100% of patients with active disease if the biopsy was obtained appropriately.^3^ Our patient’s DIF results returned positive before all other laboratory and histopathology results were available, leading to an initial presumptive diagnosis of pemphigus vulgaris that was redacted once *T*. *pallidum* staining was noted on biopsy and serologic confirmation obtained.

There have been other reports of secondary syphilis mimicking pemphigus clinically^4^^,^^5^ but, to our knowledge, this is the first case of secondary syphilis with positive DIF results for pemphigus vulgaris. Additionally, it is rare for syphilis to present with acantholysis, limited to few case reports in the literature. Of note, a case series by Kim et al highlights a vesiculopustular eruption with desquamation in newborns infected with syphilis. In this series, the newborn’s dermatopathology results demonstrated acantholysis with an eosinophilic infiltrate.^1^

Similar findings were found in a case reported by Kopelman et al who reported an 18-year-old man with pemphigus-like secondary syphilis in 2019. Like our case, he also had blurry vision and diffuse eroded erythematous scaly plaques with scalloped borders alongside vesicles and bullae. Interestingly, this patient’s biopsy results also showed keratinocyte acantholysis with eosinophilic infiltrate.^4^ Our findings support Kopelman et al’s suggestion that this same disease process that occurs in newborns could be taking place in adults as well, resulting in a similar disease presentation. Nonetheless, in contrast with Kopelman et al’s report, our patient’s DIF was positive. This case adds to the complexity of the presentation of syphilis and should prompt clinicians to add syphilis to the differential diagnosis for pemphigus-like rashes even in the setting of positive DIF staining.

## Conflicts of interest

None disclosed.
